# Patients with coronary artery disease and diabetes need improved management: a report from the EUROASPIRE IV survey: a registry from the EuroObservational Research Programme of the European Society of Cardiology

**DOI:** 10.1186/s12933-015-0296-y

**Published:** 2015-10-01

**Authors:** Viveca Gyberg, Dirk De Bacquer, Guy De Backer, Catriona Jennings, Kornelia Kotseva, Linda Mellbin, Oliver Schnell, Jaakko Tuomilehto, David Wood, Lars Rydén, Philippe Amouyel, Jan Bruthans, Almudena Castro Conde, Renata Cifkova, Jaap W. Deckers, Johan De Sutter, Mirza Dilic, Maryna Dolzhenko, Andrejs Erglis, Zlatko Fras, Dan Gaita, Nina Gotcheva, John Goudevenos, Peter Heuschmann, Aleksandras Laucevicius, Seppo Lehto, Dragan Lovic, Davor Miličić, David Moore, Evagoras Nicolaides, Raphael Oganov, Andrzej Pająk, Nana Pogosova, Zeljko Reiner, Martin Stagmo, Stefan Störk, Lale Tokgözoğlu, Dusko Vulic, On behalf of the EUROASPIRE Investigators

**Affiliations:** Cardiology Unit, Department of Medicine, Karolinska Institutet, Karolinska University Hospital Solna, 171 76 Stockholm, Sweden; Centre for Family Medicine, Department of Neurobiology, Care Sciences and Society, Karolinska Institutet, Huddinge, Sweden; European Society of Cardiology, Les Templiers, 2035 route des Colles, CS 80179 BIOT, 06903 Sophia Antipolis Cedex, France; Department of Public Health, Ghent University, Ghent, Belgium; Department of Cardiovascular Medicine, National Heart and Lung Institute, Imperial College London, London, UK; Forschergruppe Diabetes e.V. at the Helmholtz Center, Munich, Germany; Centre for Vascular Prevention, Danube-University Krems, Krems, Austria; Department of Chronic Disease Prevention, National Institute for Health and Welfare, Helsinki, Finland; Instituto de Investigacion Sanitaria del Hospital Universario LaPaz (IdiPAZ), Madrid, Spain; Diabetes Research Group, King Abdulaziz University, Jeddah, Saudi Arabia; Institut Pasteur de Lille, Inserm U744, Université Lille Nord de France, 1 rue du Professeur Calmette B.P. 245, 59019 Lille, France; Center for Cardiovascular Prevention, 1st School of Medicine, Charles University and Thomayer Hospital, Vídeňská 800, 140 59 Prague, Czech Republic; Cardiac Rehabilitation Unit, Cardiology Department, Hospital Universitario La Paz, Madrid, Spain; Thoraxcenter’s Department of Cardiology, Dr Molewaterplein 50, 3000 DR Rotterdam, The Netherlands; Department of Internal Medicine, University of Ghent, De Pintelaan 185, 9000 Ghent, Belgium; Clinical Center University of Sarajevo, Bolnička 25, 71000 Sarajevo, Bosnia and Herzegovina; Department of Cardiology of Shupyk’s Medical Academy of Postgraduate Education, 9 Dorohozhyts’ka str, Kiev, 04112 Ukraine; University of Latvia, Pauls Stradins Clinical University Hospital, Pilsonu Street 13, Riga, 1002 Latvia; Preventive Cardiology Unit, Division of Internal Medicine, University Medical Centre Ljubljana, Zaloška 7, 1525 Ljubljana, Slovenia; Medical Faculty, University of Ljubljana, Vrazov trg 2, 1000 Ljubljana, Slovenia; Institutul de Boli Cardiovasculare, Universitatea de Medicina si Farmacie “Victor Babes”, Timisoara, Romania; Department of Cardiology, National Heart Hospital, 65, Konyovitsa, 1309 Sofia, Bulgaria; Cardiology Department of Medical School, University of Ioannina, Ioannina, Greece; Institute of Clinical Epidemiology and Biometry, University of Würzburg, Würzburg, Germany; Comprehensive Heart Failure Center, University of Würzburg, Würzburg, Germany; Clinical Trial Center Würzburg, University Hospital Würzburg, Josef-Schneider-Str. 2, 97080 Würzburg, Germany; Clinic of Cardiovascular Diseases of Vilnius University, Santariskiu 2, 08661 Vilnius, Lithuania; Heart and Vascular Medicine of Vilnius University Hospital Santariskiu Clinics, Santariskiu 2, 08661 Vilnius, Lithuania; Kuopio University Hospital, Rakennus 5/6. Kerros, Puijonlaaksontie 2, 70210 Kuopio, Finland; Clinic for Internal Medicine Intermedica, Jovana Ristica 20/2, 18000 Nis, Serbia; University of Zagreb School of Medicine and University Hospital Centre Zagreb, Kispaticeva 12, HR-10000 Zagreb, Croatia; The Adelaide and Meath Hospital, Tallaght, Dublin 24, Ireland; University of Nicosia Medical School, Nicosia General Hospital, 2029 Strovolos, Nicosia, Cyprus; National Research Center for Preventive Medicine of the Ministry of Healthcare of the Russian Federation, 10 Petroverigsky per, 101990 Moscow, Russia; Department of Epidemiology and Population Studies, Faculty of Health Sciences, Jafiellonian University Medical College, Grzegórzecka 20, 31-531 Cracow, Poland; Federal Health Centre and Department of Chronic Noncommunicable Diseases Prevention, National Research Centre for Preventive Medicine, 10 Petroverigsky per, 101953 Moscow, Russia; University Hospital Centre Zagreb, School of Medicine, University of Zagreb, Kišpatićeva 12, 10000 Zagreb, Croatia; Department of Heart failure and Valve Disease, Skåne University Hospital, Lund, Sweden; Comprehensive Heart Failure Centre and Department of Medicine I, University of Würzburg, Straubmühlweg 2a, 97078 Würzburg, Germany; Hacettepe University, 06690 Ankara, Turkey; Centre for Medical Research, School of Medicine, University of Banja Luka, Vuka Karadzica 6, 78000 Banja Luka, Republic of Srpska Bosnia and Herzegovina

**Keywords:** Coronary artery disease, Type 2 diabetes, Secondary prevention, Management, Guideline adherence, Blood lipids, Blood pressure, Glycaemic control

## Abstract

**Background:**

In order to influence every day clinical practice professional organisations issue management guidelines. Cross-sectional surveys are used to evaluate the implementation of such guidelines. The present survey investigated screening for glucose perturbations in people with coronary artery disease and compared patients with known and newly detected type 2 diabetes with those without diabetes in terms of their life-style and pharmacological risk factor management in relation to contemporary European guidelines.

**Methods:**

A total of 6187 patients (18–80 years) with coronary artery disease and known glycaemic status based on a self reported history of diabetes (previously known diabetes) or the results of an oral glucose tolerance test and HbA1c (no diabetes or newly diagnosed diabetes) were investigated in EUROASPIRE IV including patients in 24 European countries 2012–2013. The patients were interviewed and investigated in order to enable a comparison between their actual risk factor control with that recommended in current European management guidelines and the outcome in previously conducted surveys.

**Results:**

A total of 2846 (46 %) patients had no diabetes, 1158 (19 %) newly diagnosed diabetes and 2183 (35 %) previously known diabetes. The combined use of all four cardioprotective drugs in these groups was 53, 55 and 60 %, respectively. A blood pressure target of <140/90 mmHg was achieved in 68, 61, 54 % and a LDL-cholesterol target of <1.8 mmol/L in 16, 18 and 28 %. Patients with newly diagnosed and previously known diabetes reached an HbA1c <7.0 % (53 mmol/mol) in 95 and 53 % and 11 % of those with previously known diabetes had an HbA1c >9.0 % (>75 mmol/mol). Of the patients with diabetes 69 % reported on low physical activity. The proportion of patients participating in cardiac rehabilitation programmes was low (≈40 %) and only 27 % of those with diabetes had attended diabetes schools. Compared with data from previous surveys the use of cardioprotective drugs had increased and more patients were achieving the risk factor treatment targets.

**Conclusions:**

Despite advances in patient management there is further potential to improve both the detection and management of patients with diabetes and coronary artery disease.

## Background

The backbone of clinical practice should be evidence derived from research and not least prospective, randomised, controlled clinical trials. An important tool for distributing contemporary knowledge is guidelines written by experts in various fields of medicine. The management of patients with coronary artery disease has since long been the subject of such guidelines.

One third of patients with coronary artery disease have diabetes, a rapidly growing disease globally [1–3]. Patients with both diagnoses have a considerably higher mortality than coronary patients without diabetes [4, 5]. A comprehensive, target driven lifestyle and pharmacological strategy improve their prognosis to be almost similar to that of patients without diabetes [6, 7]. Still preventive management of cardiovascular patients, especially those with diabetes, remains inadequate [6–8].

The European Society of Cardiology has issued guidelines on cardiovascular prevention in clinical practice since 1994 with the aim to improve the practice of preventive cardiology. The EUROASPIRE (European Action on Secondary and Primary Prevention by Intervention to Reduce Events) cross sectional surveys have since 1995, evaluated the implementation of these guidelines [9]. A similar survey, the EuroHeart Survey on Diabetes and the Heart [7] focused on patients with diabetes. Educational activities and updates of guidelines have been based on the outcome of these surveys with the intention to further improve every day practice. A follow up of the outcome of these efforts is important to provide feed back on aspects of cardiovascular prevention in need of further refinement.

The aim of the present investigation was to study screening for glucose perturbations in people with coronary artery disease and to compare life-style and pharmacological risk factor management in patients with known and newly detected type 2 diabetes with those without diabetes in relation to European Guidelines on prevention and the management of diabetes and its prestates issued in 2007, 2012 and 2013 [9–11].

## Methods

EUROASPIRE IV was conducted at 79 centres in 24 European countries during May 2012 to April 2013 [12]. Men and women aged ≥18 to <80 years were identified by the following diagnoses of first or recurrent coronary artery disease at a time 6–36 months prior to the present investigation: (1) coronary artery bypass grafting (CABG), (2) percutaneous coronary intervention (PCI), (3) acute myocardial infarction (AMI), and (4) acute myocardial ischemia.

Information on personal and demographic details, self reported lifestyle and medication were obtained during an outpatient visit at the participating centres. Data collectors were trained to use standardized methodologies for physical measurements.

The following variables were recorded:

*Height and weight* were measured in light indoor clothes without shoes (scales 701 and measuring stick model 220; SECA Medical Measuring Systems and Scales, Birmingham, UK).

*Waist circumference* was measured using a metal tape applied horizontally at the point midway in the mid-axillary line between the lowest rim of the rib cage and the tip of the hip bone (superior iliac crest) with the patient standing [13]. Central obesity was defined as a waist circumference of ≥88 cm for women and ≥102 cm for men.

*Blood pressure* was measured twice on the right upper arm in the sitting position using an automatic digital sphygmomanometer (Omron M6; OMRON Corporation, Kyoto, Japan) and the mean was used for the analyses.

*Physical activity* was assessed by means of the International Physical Activity Questionnaire (IPAQ; IPAQ core group, Karolinska Institutet, Stockholm, Sweden).

*Smoking* at the time of interview was defined as self-reported smoking, and/or a breath carbon monoxide exceeding 10 ppm (Bedfont Scientific, Model Micro+) [14].

*Blood lipids* were measured in the fasting state and analysed at the central laboratory (Disease Risk Unit, National Institute for Health and Welfare, Helsinki, Finland) on a clinical chemistry analyser (Abbot Architect analyzer; Abbott Laboratories, Abbott Park, IL, USA) using enzymatic method for measuring total cholesterol.

*Glycated haemoglobin A1c* (*HbA1c*) was measured at a central laboratory with an immunoturbidimetric method (Abbot Architect analyzer; Abbott Laboratories, Abbott Park, IL, USA) in fasting venous whole blood sampled in an EDTA-tube.

*An oral glucose tolerance test* (*OGTT*) was performed using 75 grams of glucose in 200 mL of water in the morning after at least 10 h of fasting. Blood for fasting plasma glucose (*FPG)* was drawn before intake of glucose with a dip safe from the EDTA-tube in which the HbA1c was collected. Samples for the 2 h post-load glucose (2hPG) measurement was drawn from whole venous blood using an EDTA-tube. Plasma glucose was analysed locally in all centres with a photometric point-of-care technique (Glucose 201, HemoCue^®^, Ängelholm, Sweden) [15]. The HemoCue^®^ method is cholesterol sensitive due to the measurement in very small volumes with higher levels of glycaemia at low cholesterol levels; therefore the glucose values were corrected for cholesterol according to the formula: HemoCue^®^ glucose + 0.22 × (total cholesterol − 5). Prior to final data analysis the values were converted from whole venous blood to plasma applying the formula established by Carstensen et al. [16]: plasma glucose = 0.558 + 0.119 × whole blood glucose. Proper use of the equipment was assured through central training of the data collectors, and retrieval of HemoCue^®^-cuvette storage information and validation sheets from a selection of the participating centres.

*The use of four cardioprotective drug therapies* consisting of antiplatelet drugs, β-blockade, renin–angiotensin–aldosterone–system (RAAS) blockade (including angiotensin converting enzyme inhibitors and angiotensin receptor blockers) and statins was assessed at the outpatient visit.

*Treatment target attainment* was assessed for blood pressure, LDL-cholesterol and HbA1c as outlined in Table 1.

**Table 1:** Treatment targets according to the European Guidelines for Diabetes, Pre-Diabetes and Cardiovascular Disease as issued 2007 [10], and updated in 2013 [11] and European Guidelines on Cardiovascular Disease Prevention in clinical practice issued 2012 [9]

Variable	Diabetes 2007	Prevention 2012	Diabetes 2013
Blood pressure (mm Hg) (no diabetes)	<140/90	<140/90	<140/90
Blood pressure (mm Hg) (diabetes)	<130/80	<140/80	<140/85
LDL-cholesterol mmol/L (mg/dL) (no diabetes and diabetes)	<1.8 (<70)	<1.8 (<70)	<1.8 (<70)^a^
HbA1c % (mmol/mol) (diabetes)	≤6.5 % (≤48)	≤7.0 % (≤53)	≤7.0 % (≤53)

^a^Or at least a ≥50 % LDL-cholesterol reduction if this target cannot be reached

*Information on health care providers* including participation in educational programmes involving physical activity, dietary advice and instructions on medications.

### Patient groups

The participants were divided into three groups based on their glycaemic state:

#### No diabetes

No history of diabetes and all three tests FPG, 2hPG and HbA1c fulfilling the following criteria: FPG <7.0 mmol/L (126 mg/dL) and 2hPG value <11.1 mmol/L (200 mg/dL) and HbA1c <48 mmol/mol (<6.5 %; Diabetes Control and Complications Trial [DCCT]-standard).

#### Newly diagnosed diabetes

No history of diabetes and at least one of the three tests FPG, HbA1c and 2hPG fulfilling the following criteria: FPG ≥7.0 mmol/L (126 mg/dL) and/or 2hPG value ≥11.1 mmol/L (200 mg/dL) and/or HbA1c ≥48 mmol/mol (≥6.5 %; DCCT standard)

#### Previously known diabetes

A self-reported history of diabetes and/or prescribed glucose lowering drugs.

### Data management

Data were collected electronically and submitted online to the data management centre (EuroObservational Research Program for EUROASPIRE IV, European Heart House, Sophia Antipolis, France). Data were checked for completeness, internal consistency and accuracy. All data were stored under the provisions of the National Data Protection Regulations.

### Statistical analyses

The distributions of patient characteristics across groups (Table 2) were compared according to the Kruskal–Wallis test and the Chi square test, respectively. Use of pharmacological treatments and healthcare providers consulted were statistically compared between groups according to multilevel logistic modelling to account for the clustering of patients within centres [17]. Distributions of blood pressures, LDL cholesterol and HbA1c levels in patients with diabetes, were compared through multilevel linear modelling. All models included age and sex as covariates, A level of alpha <0.05 was a priori chosen to indicate statistical significance. All statistical analyses were performed at the Department of Public Health, Ghent University, Belgium by means of SAS statistical software release 9.3 (SAS Institute Inc., Cary, NC, USA).

**Table 2:** Patient characteristics of the 6187 included and 1811 excluded patients

Variable	Diabetes			*P* value (difference between groups)	Missing info n = 1811
	No n = 2846	Newly diagnosed n = 1158	Previously known n = 2183		
Participants (%)	46	19	35		
Age (years)					
Mean (SD)	63 (10.0)	65 (9.2)	65 (8.6)	<0.0001	64 (9.6)
<50	320 (11)	68 (6)	103 (5)		191 (11)
50–59	714 (25)	263 (23)	451 (21)		492 (27)
60–69	1026 (36)	437 (38)	900 (41)		622 (34)
>70	786 (28)	390 (34)	730 (33)		506 (28)
Sex				0.006	
Women	674 (24)	268 (23)	594 (27)		412 (23)
Men	2172 (76)	890 (77)	1589 (73)		1399 (77)
Smoker	464 (16)	165 (14)	304 (14)	0.04	346 (19)
Body mass index, kg/m	28 (4.2)	29 (4.5)	31(5.0)	<0.0001	29 (4.7)
<25	638 (23) (n = 2841)	164 (14)	239 (11) (n = 2171)		384 (21) (n = 1792)
25–29.9	1349 (48) (n = 2841)	533 (46)	846 (39) (n = 2171)		818 (46) (n = 1792)
≥30	854 (30) (n = 2841)	461 (40))	1086 (50) (n = 2171)		590 (33) (n = 1792)
Central obesity	1439 (51) (n = 2806)	724 (63) (n = 1146)	1510 (71) (n = 2135)	<0.0001	895 (51) (n = 1768)
Low or moderate physical activity (IPAQ questionnaire)	1215 (55) (n = 2214)	484 (54) (n = 901)	1079 (69) (n = 1667)	<0.0001	754 (57) (n = 1318)
FPG mmol/L [mean (SD)]	6.0 (0.6)	7.3 (0.9)	8.6 (2.9)	<0.0001	6.1 (1.7)
2hPG mmol/L [mean (SD)]	7.1 (1.7)	10.1 (3.4)	–	<0.0001	–
HbA1c mmol/mol [mean (SD)]	5.6 (0.3)	6.0 (0.6)	7.2 (1.4)	<0.0001	5.8 (0.7)

Data presented are n (%) if not stated otherwise. In case of missing data the total number of observations is given in below the n (%) information

### Ethics

National Co-ordinators were responsible for obtaining Local Research Ethics Committees approvals. Written, informed consent was obtained from each participant by the investigator by means of a signed declaration. The research assistants signed in the Case Record Form to confirm that informed consent was obtained and stored the original of the signed declaration of consent in the patient file.

## Results

A total of 16,426 medical records were reviewed in the search for study participants and 7998 (49 %) attended the interview. The reasons for 8428 non-participants were as follows: no response to the invitation letter (35 %), refusal to attend for personal reasons (39 %), changing medical condition or deceased (16 %), living outside the catchment area (3 %) and miscellaneous (7 %). The median time between the index event and the interview was 16 months (interquartile range 12–22 months). Full information on the glycaemic state was available in 6187 (77 %) of the participants who attended the interview out of whom 2846 (46 %) had no diabetes, 1158 (19 %) had newly diagnosed diabetes and 2183 (35 %) had previously known diabetes, the vast majority with type 2 and only 41 with type 1 diabetes. Clinical characteristics of the patients with (n = 6187) and without (n = 1811) known glycaemic state at the time of interview are presented in Table 2.

**Fig. 1:**
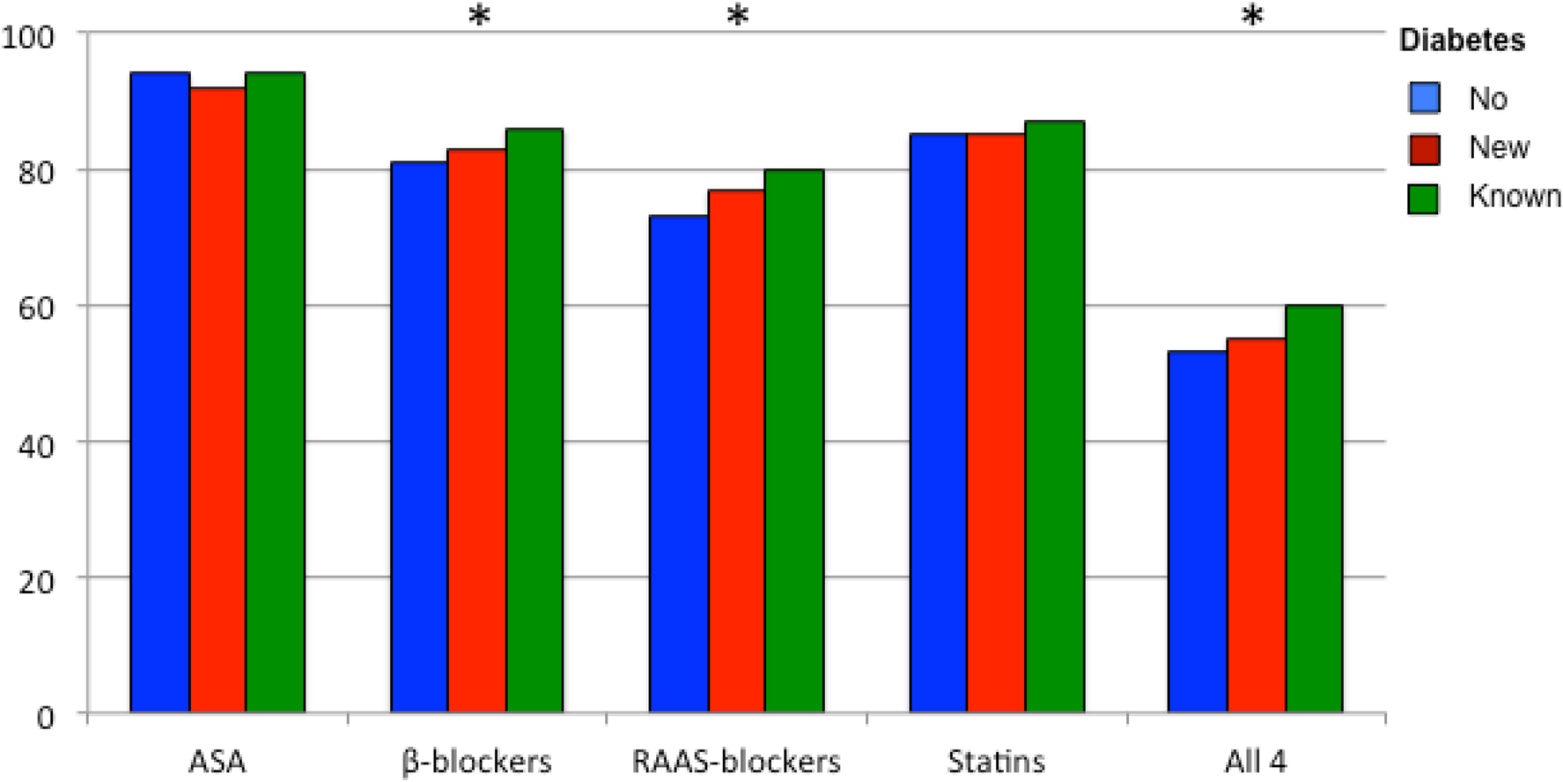
Proportion of patients with no, newly diagnosed and known diabetes prescribed the different cardioprotective drugs alone and in combination. Renin–angiotensin–aldosterone–system (RAAS)-blockers includes angiotensin converting enzyme inhibitors and angiotensin receptor blockers. *ASA* aspirin. All 4 = the combination of aspirin (or other anticoagulants) + a β-blocker + a RAAS-blocker and a statin. *p < 0.0001

There were small differences between the three groups regarding the use of antiplatelet agents (predominantly aspirin), β-blockers, RAAS-blockers and statins. A combination of all four cardioprotective drug classes was prescribed to ≤60 % of the patients (Fig. 1).

### Glucose lowering treatment

Sixty five per cent of the patients with previously known diabetes had been given dietary advice. The most commonly used glucose lowering drugs in patients with previously known diabetes were metformin (57 %), insulin (27 %), sulfonylurea (25 %) and DPP-4 inhibitor (7 %). The most common combinations of glucose lowering drugs were metformin + sulfonylurea (15 %), metformin + insulin (11 %) and metformin + DPP-4 inhibitor (6 %). The vast majority of patients (97 %) in all three groups responded that they seldom or never altered or missed their medication.

### Target achievement

**Fig. 2:**
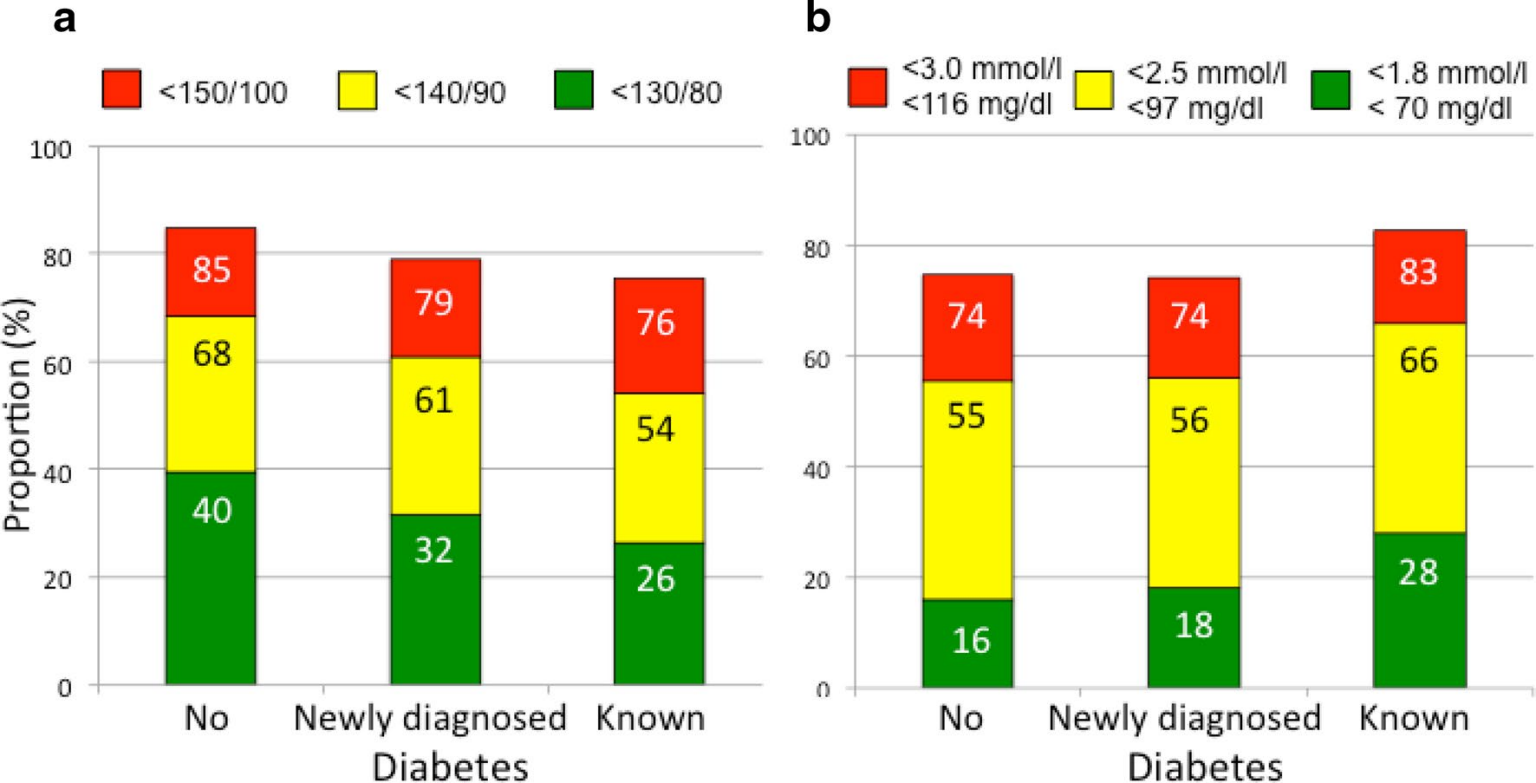
**a** Proportion of patients with no, newly diagnosed and known diabetes reaching different blood pressure targets. **b** Proportion of patients with no, newly diagnosed and known diabetes reaching different LDL-cholesterol targets.

The proportion of patients with no, newly diagnosed and previously known diabetes reaching a blood pressure target of <150/100, <140/90 mmHg (target for patients with no diabetes 2012 [9]) and the target of <130/80 mmHg (target for patients with diabetes 2007 [10]) is presented in Fig. 2a. A blood pressure below 110 mm Hg was observed among 5 % of the patients.

The corresponding proportions of the three patient categories reaching the LDL-cholesterol target of <1.8 mmol/L (target for all patients with established cardiovascular disease 2007 [10], 2012 [9] and 2013 [11]) is presented in Fig. 2b. The LDL-cholesterol exceeded 3.0 mmol/L in 26, 26 and 17 % of patients with no, newly diagnosed and previously known diabetes, respectively. Total cholesterol <4.5 mmol/L (target for all patients with cardiovascular disease 2007 [10] and 2013 [11]) was reached in 59, 59 and 68 %. Patients with newly diagnosed and previously known diabetes had HbA1c <7.0 % (53 mmol/mol) in 95 and in 53 %, respectively. The proportion of patients with previously known diabetes that had an HbA1c >9.0 % (>75 mmol/mol) was 11 %.

### Health care providers

An overview of healthcare providers is presented in Table 3. Twenty seven per cent of the patients with previously known diabetes had attended a diabetes clinic.

**Table 3:** Health-care provider delivering the care presented as % (n/total n) multiple health-care providers are possible explaining that columns add >100 %

Health-care provider	Diabetes			
	No	Newly diagnosed	Previously known	P value^a^
General practitioner	58 (1662/2845)	62 (717/1158)	65 (1408/2183)	0.07
Cardiologist	67 (1916/2846)	73 (848/1158)	74 (1618/2183)	0.69
Endocrinologist/diabetologist	1 (40/2846)	2 (18/1158)	34 (731/2183)	<0.0001
Cardiac specialist nurse	5 (134/2846)	3 (30/1158)	5 (114/2183)	0.001
Cardiac rehabilitation program	43 (1201/2824)	39 (451/1145)	36 (777/2154)	<0.0001

^a^Taking into account clustering of patients within centres and adjusted for age and sex

## Discussion

The three main findings in this first survey evaluating whether European Guidelines for Diabetes, Prediabetes and Cardiovascular Disease in 2007 [10] had an impact on the management of such patients are: (1) about one fifth of the participants had previously undetected diabetes. The failure to screen for diabetes when these coronary patients were first seen, 1–2 years before the interview, led to a lost opportunity to institute early and effective preventive measures in a particularly vulnerable group of patients; (2) although the combined use of four cardioprotective drug classes compares favourably with the proportions seen in previous surveys, a considerable proportion of the present patient population still does not reach guideline recommended treatment targets of blood pressure, blood lipids and of glycaemic control; (3) life style adaptation was poor among many patients as exemplified by a majority reporting low physical activity and in general low participation in cardiac rehabilitation programmes and diabetes clinics for those with known diabetes. It can therefore be stated that even if the 2007 European guidelines had some impact on patient management there is still a considerable potential to reduce the cardiovascular risk in patients with coronary artery disease and diabetes just by adhering to available knowledge as reflected in management guidelines, the most recent issued in 2013 [11].

The proportions of patients with newly diagnosed (19 %) and previously known diabetes (35 %) were similar to those reported in EuroHeart Survey of Diabetes and the Heart (14 and 31 %, respectively). Guideline recommendation is to screen systematically for diabetes in all coronary patients without self-reported diabetes [11]. This recommendation was not followed at the time for the index coronary event. In the present cohort a vast majority of the patients with newly diagnosed diabetes had an HbA1c of <6.5 % (<48 mmol/mol) underlining the need for an OGTT if FPG and HbA1c are negative as recently reported [18].

Blood pressure control was better compared to EUROASPIRE III and best in patients without diabetes. The proportions of patients with no diabetes, newly diagnosed diabetes and previously known diabetes achieving a target of <140/90 mm Hg was 52, 49 and 43 % in EUROASPIRE III [8] and 68, 61 and 54 % in the present survey. Both surveys reflect the difficulties in controlling blood pressure in patients with diabetes, who often require a combination of several different blood pressure lowering drugs. Even if patients with diabetes had the highest use of both RAAS-blockers and β-blockers it seems that their antihypertensive therapy needs to be strengthened [19]. Insufficient patient compliance may have contributed even if a majority of patients denied such behaviour. Unfortunately the available information does not allow a more detailed analysis of this possibility. An unexpected, but important, observation is that a group of patients had blood pressure levels well below guideline recommended targets. This finding should induce caution in consideration of the J-shaped curve of blood pressure control, with increasing cardiovascular events among coronary patients with a blood pressure consistently below the recommended level [20].

Of patients with no, newly diagnosed and previously known diabetes 12, 11 and 20 % reached a LDL-cholesterol target of <1.8 mmol/L in EUROASPIRE III [8]. The corresponding proportions were 16, 18 and 28 %. Anyhow lipid management could undoubtedly have been further improved given that the target of <1.8 mmol/L was recommended already 5–6 years before the initiation of the present survey [10]. As demonstrated by the Euro Heart Survey on Diabetes and the Heart [6] and the STENO 2 trial [21] adequate blood pressure and LDL-cholesterol control is of critical importance for patients with diabetes. From the latter study it emerged that lipid control accounted for 73 % of the observed decrease in morbidity and mortality while the corresponding impact of blood pressure and glucose control was 11 and 13 %, respectively [22]. Furthermore, lipid control is important not only for macro- but also microvascular complications in patients with diabetes [23, 24].

Glycaemic control in patients with previously known diabetes was similar to that observed in EUROASPIRE III [8, 25]. Considering the risk of micro- and macrovascular complications [26, 27] it is of great concern that 10 % of patients with known diabetes had an HbA1c >9.0 % (75 mmol/mol). It has become apparent from randomised trials that the reduction of macrovascular events by glucose-lowering strategies has yet to be proven and may only be seen after many years of follow-up [21, 28].

The rationale for not only reporting the total proportion of patients above the presently recommended target but also at more elevated levels was to show that large proportions have considerably higher cholesterol, blood pressure and glucose levels and to verify that the present findings were not related to patients being just above the recommended target.

There was no difference between EUROASPIRE III and IV in how many patients had a cardiologist as their health-care provider but the proportion of patients seen by a general practitioner was higher as was the proportion of patients with previously known diabetes seen by an endocrinologist. This changing pattern of caregivers may at least explain, to some extent, the improved management. On the negative side only 3–5 % of the present patient cohort attended nurse led prevention programmes and less than one third of patients with diabetes had been offered specialist diabetes education.

A likely contributor to the observed improvement is the emphasis on the poor prognosis for patients with a combination of diabetes and coronary heart disease as reflected in the first Joint European Guidelines on diabetes, pre-diabetes and cardiovascular diseases issued in 2007 [10]. In an evaluation of the use of these guidelines almost nine out of 10 physicians, mainly general practitioners and internists, had the guidelines at their work place, and eight out of 10 were aware of and applied them in their daily practice [29].

Diabetes schools and comprehensive cardiovascular rehabilitation is achievable and efficient and has the potential to improve treatment given patient participation [9, 30]. The under use of such programmes is a plausible explanation for the poor control of life-style oriented factors with a high proportion of obesity, low physical activity in a majority, and an unfortunate 14–16 % of the patients still smoking. Moe et al. [31] recently studied the impact of physical activity on cardiovascular mortality in a large cohort of people with and without diabetes followed during 24 years. Inactive people with diabetes, especially those on medical treatment, had a significantly increased mortality risk compared with those who reported taking weekly physical exercise of at least 2 h. This underlines the seriousness of the present findings indicating inertia and neglected efforts to improve life-style habits not least physical activity.

### Strengths and limitations

Participating centres included university teaching hospitals, specialist cardiac centres and some district hospitals. Volunteer participation in a survey may indicate a particular interest in cardiovascular prevention. This bias is conservative as the standard of care in participating centres interested in prevention is likely to be high while care of patients in everyday practice is probably worse. The average interview rate was rather low, 49 %. Usually non-participants are more likely to have unhealthy lifestyles and the present data, if anything, may overestimate the true standard of preventive medicine across Europe. As can be seen in Table 2 the patients without full glycaemic information were more similar to those with no diabetes, which may have led to some overestimation of the proportions of known and newly detected diabetes. The fact is unlikely to affect the main conclusions of this survey considering the high prevalence of patients with glucose perturbations. The centres and countries participating in European Heart Survey of Diabetes and the Heart, EUROASPIRE III and IV are not the same, which has to be kept in mind when interpreting time trends. For logistic reasons only one sample of FPG, 2hPG and HbA1c was collected. A definite diagnosis of diabetes should be based on at least two measurements. Finally very few patients (n = 41) had type 1 diabetes making meaningful comparisons between them and those with type 2 diabetes impossible.

A main strength of EUROASPIRE IV is that data are based on interviews and standardized examinations rather than data from medical records, and that the survey despite a seemingly low participation rate provides comparative information on preventive care in a large cross-sectional European population of well-characterized individuals with coronary artery disease. The sample size allowed a statistically robust comparison between groups according to their glycaemic state. All three tests—FPG, 2hPG and HbA1c—as currently recommended for the classification of patients into their respective glycaemic category, were used thereby avoiding misclassification.

## Conclusion

The prevalence of self reported diabetes underestimates the true prevalence of diabetes in coronary patients. A systematic approach to screen for diabetes in coronary patients is required. Although some of the risk factors studied were better controlled than in previous surveys, there is still considerable potential to further reduce cardiovascular risk through lifestyle and optimised cardioprotective medications in patients with diabetes and coronary artery disease through implementation of knowledge into every day clinical practice. This is particularly so for LDL-cholesterol where a large majority of patients do not reach the treatment target of <1.8 mmol/L but blood pressure and glycaemic control also need further refinement.
